# Gene expression profiles and physiological data from mice fed resveratrol-enriched rice DJ526

**DOI:** 10.1038/sdata.2016.114

**Published:** 2016-12-20

**Authors:** Hea-Jong Chung, Heui-Kwan Lee, Hyeon-Jin Kim, So-Hyeon Baek, Seong-Tshool Hong

**Affiliations:** 1 Department of Biomedical Sciences and Institute for Medical Science, Chonbuk National University Medical School, Jeonju, Chonbuk 54907, South Korea; 2 Deparment of Radiation oncology, Presbyterian Medical Center, Seonam University Medical School, Jeonju, Chonbuk 54987, South Korea; 3 JINIS BDRD institute, JINIS Biopharmaceuticals Co., 948-9 Dunsan, Bongdong, Wanju, Chonbuk 55321, South Korea; 4 Department of Well-being Resources, Sunchon National University, Suncheon, Jeonnam 57922, South Korea

**Keywords:** Reverse transcription polymerase chain reaction, Ageing, Microarray analysis, Mouse

## Abstract

The molecular mechanism underlying lifespan extension by resveratrol remains widely discussed. To help study this mechanism, we previously created resveratrol-enriched rice, DJ526, by transferring the resveratrol biosynthesis gene into Dongjin rice. DJ526 accumulates 1.4–1.9 μg g^−1^ of resveratrol in its grain and can ameliorates age-related deterioration in mice, as compared to control animals, based on assessments of motor coordination, physical strength and cutaneous tissue aging. Here, we present raw data sets, deposited in public repositories, from microarray analysis and physiological data of mice fed with DJ526 and Dongjin rice and treated with resveratrol. We also provide a method to analyze blood serum at micron levels. These data sets may help other researchers find new clues regarding the etiology of the anti-aging process and signaling pathways induced by resveratrol, rice, or DJ526.

## Background & Summary

Resveratrol has gained widespread attention because of its ability to extend lifespan in some model organisms, and to protect against age-related diseases, such as cancer, Alzheimer’s, and diabetes in mammals^1^. Lifespan extension by resveratrol has been observed in yeast, worms, fruit flies, and fish^2–8^. In rodents, however, resveratrol has been shown to produce changes associated with longer lifespan, but does not affect lifespan itself^9^. A small molecule that activates SIRT1, in a manner similar to resveratrol, was shown to extend the lifespan of mice on a normal diet^10^. Thus far, the beneficial effect of resveratrol in higher animals, including humans, appears to be limited to mimicking dietary restriction to delay the physiological deterioration associated with aging and not extending lifespan *per se*^11–15^.

Previously, we showed that resveratrol-enriched rice DJ526, developed by transferring the resveratrol biosynthesis gene, stilbene synthase, from the peanut *Arachis hypogaea* variety Palkwang into the rice *Oryza sativa japonica* variety Dongjin, shows beneficial effects by mitigating metabolic syndrome and obesity^16,17^. We hypothesized that the resveratrol-enriched rice DJ526, which accumulates 1.4–1.9 μg g^−1^ of resveratrol in its grain, could show comparable health benefits to those shown by crops containing similar resveratrol levels. Notably, DJ526 acted synergistically with the transgenic resveratrol trait to confer unexpectedly higher health benefits on experimental mice than those expected from mice feeding on either Dongjin rice or being treated with resveratrol. Specifically, genetically modified DJ526 rice was able to treat metabolic syndrome and obesity in mice, even though Dongjin rice and resveratrol on their own showed no significant effect^16,17^. The efficacy levels of DJ526 in ameliorating metabolic syndrome and obesity were comparable to the typical pharmaceutical drugs designed to treat metabolic syndrome and obesity^16,17^.

We have previously published DJ526’s beneficial effects on aging, as well as the underlying mechanism of action^18^. DJ526 ameliorated age-related deterioration in mice, as demonstrated by inhibition of cutaneous tissue aging, and a boost in motor coordination and physical strength, whereas Dongjin rice or resveratrol on their own showed insignificant effects on age-related deterioration^18^.

In the present study, we performed whole-genome microarray, biosynthetic pathway, and physiological analyses using liver samples of mice fed with the resveratrol-enriched rice DJ526, Dongjin rice, and resveratrol on its own. Results of these data analyses were published in *Scientific Reports*, focusing on the interpretation of data, as well as elucidating the physiological impact of feeding mice with DJ526, Dongjin rice, and resveratrol^18^. In this manuscript, we describe the raw data from our microarray, blood serum and behavioral assays, which have each been deposited in public repositories. For decades, resveratrol has been one of the most debated compounds for their health-beneficial effects. Considering these characteristics of resveratrol and DJ526, we believe that our microarray data could be a valuable resource in studying the process of aging and elucidating the underlying mechanism of resveratrol’s health benefits.

Mice blood samples are routinely required for a wide variety of *in vivo* experiments. Although a number of efficient methods have been developed, current methods typically require sacrificing mice or exposing them to considerable stress^19^. It is known that the outcome of research experiments is affected if mice are exposed to stress during blood collection^20^. Therefore, we developed a method to analyze mouse blood at the micron scale, whereby minuscule amount of blood was collected in a short period and analyzed accurately to avoid inducing significant stress.

## Methods

### Study design

**Figure 1:**
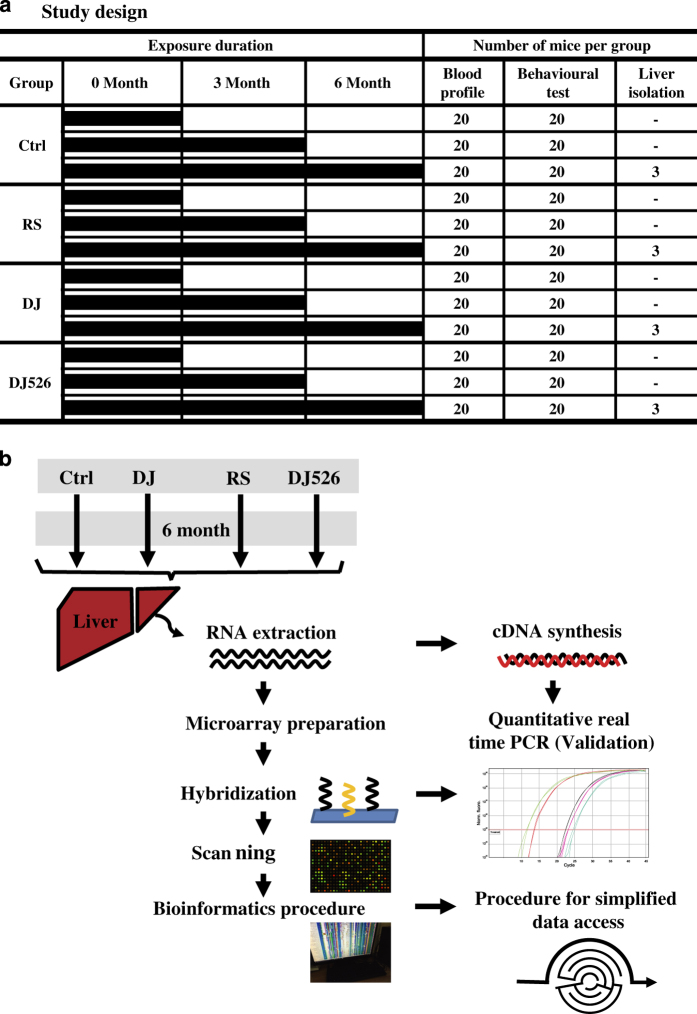
Experimental design. (**a**) The study design entailed four different exposure groups and three time points (0, 3, and 6 months), where mice were allocated for each category. The number of mice assigned to each exposure group for each time point and design are summarized. (**b**) Schematic illustration of microarray analysis. For detailing gene expression patterns of the four mouse groups, we performed whole-genome microarray and pathway analyses using the liver samples of mice fed with the resveratrol-enriched rice DJ526 (DJ526), Dongjin rice (DJ), treated with resveratrol alone (RS), and control (Ctrl).

The study design included four groups of female C57BL/6N mice (*n*=80; 20 per group) (1) Ctrl (control mice fed with NFD in which the carbohydrate sources were corn starch and sucrose); (2) RS (resveratrol-treated mice fed with NFD wherein the carbohydrate sources were corn starch and sucrose except containing resveratrol); (3) DJ (Dongjin mice fed a NFD in which the corn starch and sucrose were replaced with Dongjin rice); and (4) DJ526 (DJ526 mice fed with NFD in which the corn starch and sucrose were replaced with the resveratrol-enriched rice DJ526) (Fig. 1a).

### Ethics approval

All procedures involving the mice were accredited with the approval of Institutional Animal Care and Use Committee (IACUC), in compliance with the guidelines of Ethics Committee of Chonbuk National University Laboratory Animal Center Guidelines on the Care and Use of Animals for Scientific Purposes. Each animal-related procedural change during the course of our study was communicated, discussed, and approved by the IACUC committee. All animal experiments in this study were in accordance with the ARRIVE guidelines (https://www.nc3rs.org.uk/arrive-guidelines) and checklist. Our ARRIVE checklist was deposited at figshare (Data Citation 1).

### Animals

Six-week-old C57BL/6N female mice (*n*=80; each weighing approximately 17.5 g±1.5 g) were purchased from Joongang Experimental Animal Co. (Seoul, South Korea). Ten animals were house in one cage, and were provided with food (10% kcal as fat intake; D12450B; Research Diets Inc., New Brunswick, NJ, USA) and water *ad libitum*, unless otherwise stated. The mice cages were randomly assigned to each of the four groups previously defined, with 20 animals per group. A 12-h light–dark cycle was maintained (lights on at 8:30 PM daily) in a room with controlled temperature (22 °C±1 °C) and humidity (55%±5%) for the animals. After 2 weeks of acclimation, 80 mice were randomly divided into the following groups: Normal Formula Diet (Ctrl), NFD supplemented with resveratrol (RS), NFD in which the cornstarch and sucrose were replaced with Dongjin rice (DJ), NFD in which the cornstarch and sucrose were replaced with the resveratrol-enriched rice DJ526 (DJ526). The composition of the diets was described previously^18^. Resveratrol concentration in all the formulized diets was quantified by HPLC (ACQUITY UPLC, Waters, MA, USA) as described previously^16,17^. Food consumption of each mouse group was monitored daily. For blood profiling and behavioral tests, animals were randomly assigned to each treatment groups. In particular, we declare that blinding was employed during animal allocation and data collection.

### Blood collection and micron-scale assays

Blood glucose and lipid levels were measured at 0, 3, and 6 months during treatment. The food consumption of each mouse group was regularly monitored. Blood was collected in 1.5 ml Eppendorf micro-centrifuge tubes by making a precise incision above the mouse-tail vein after 5 h of starvation. Blood serum was separated by centrifuging at 13,000 rpm for 10 min and was immediately stored at 22 °C until assayed. If serum quantity was too small even in micro-scale, assay readings would not be accurate. Therefore, two serum samples from within the same group were randomly combined to acquire sufficient serum volume for analyses. Blood glucose levels were measured using an Accu-check Glucometer (Roche, Basel, Switzerland). Serum from each mouse group (1–2 μl) was transferred to 96-well microplates to measure triglyceride, total cholesterol, and HDL-cholesterol levels. Recommended amounts of the reagents for the quantification, which were reduced proportionally based on manufacturer instructions (Asan Pharmaceutical, Seoul, Korea), were added into each well, and quantification was performed using enzymatic colorimetric methods. Raw data of the blood profiles was deposited at figshare (Data Citation 1).

### Mouse behavioral test

Each group of mice was tested at 0, 3, and 6 months after the treatment regime began to assess their behavior, which was also described in our previous publication^17^. The animals’ swimming, motivation, and coordination ability was assessed by forced swim test (FST); rotarod (ROTA ROD, Haryana, India) performance test was also performed to test their coordination, balance, and neuro-muscular strength^21,22^.

Before starting FST, the mice were trained to swim from one end of a water-filled glass tank to a visible escape platform, located 0.5 cm below the water surface, at the opposite end. The glass tank (40×25×16 cm^3^) was filled to a depth of 20 cm, with the water maintained at 23 °C. After training, the mice were released from one end during each test trial, and were allowed to swim freely for up to 120 s. If an animal successfully reached the hidden platform within 120 s, it was allowed to remain there for 5 s and was then removed from the tank. If an animal failed to find the platform within 120 s, it was manually placed onto the platform and was allowed to remain there for 5 s. No spatial cues were provided within the tank environment.

The rotarod apparatus (ROTA ROD, Haryana, India) was used to assess motor coordination, strength, and balance. The rotarod apparatus consisted of a base platform and a rotating rod with a diameter of 3 cm and a non-slippery surface. The rod was placed at a height of 15 cm from the base. The rod, 30 cm in length, was divided into four equal sections by three fiber plates. Therefore, up to four mice were tested simultaneously on the apparatus, with the same rotating speed of the rod. The four mouse groups were tested over three consecutive days. Each daily session included a single training trial of 5 min at 4 RPM on the rotarod apparatus. One hour later, the animals were tested for three consecutive accelerating trials of 5 min with the rotarod speed changing from 0 to 60 RPM over 300 s, with an inter-trial interval of at least 30 min. The mean latency to fall off the rotarod was recorded and used in the subsequent analysis. The unprocessed behavioral raw data of the FST and rotarod test were deposited at figshare (Data Citation 1).

### Microarray assay

Three mice per group at 6 months after start of treatment course were sacrificed, and their livers were harvested and flash frozen. Approximately 100 mg of each liver was used for RNA extraction by RNeasy Mini kit in accordance with the manufacturer protocol (Qiagen, Limburg, Netherlands), and RNA-extraction quality was checked (Table 1). For control and test RNA extraction, the synthesis of target cRNA probes and hybridization was performed using Agilent’s Low RNA Input Linear Amplification kit (Agilent Technology, CA, USA) according to the manufacturer’s instructions. Briefly, each 1 μg of total RNA and T7 promoter primer mix were incubated at 65 °C for 10 min. cDNA master mix (5× First strand buffer, 0.1 M DTT, 10 mM dNTP mix, RNase-Out, and MMLV-RT) was prepared and added to the reaction mixer. The samples were incubated at 40 °C for 2 h, and then RT and dsDNA synthesis was terminated by incubating at 65 °C for 15 min. The transcription master mix was prepared according to the manufacturer’s protocol (4× Transcription buffer, 0.1 M DTT, NTP mix, 50% PEG, RNase-Out, Inorganic pyrophosphatase, T7-RNA polymerase, and Cyanine 3-CTP). Transcription of dsDNA was performed by adding the transcription master mix to the dsDNA reaction samples and incubating at 40 °C for 2 h. Amplified and labeled cRNA was purified using cRNA Cleanup Module (Agilent Technology, CA, USA) according to the manufacturer’s protocol. Labeled cRNA targets were quantified using ND-1000 spectrophotometer (NanoDrop Technologies, Inc., Wilmington, DE). After checking cRNA-labeling efficiency (Table 2), fragmentation of cRNA was performed by adding 10× blocking agent, 25× fragmentation buffer, and incubating at 60 °C for 30 min. The fragmented cRNA was resuspended with 2× hybridization buffer and was directly pipetted onto assembled Agilent’s Mouse Gene Expression 4X44K v2. Hybridization was performed at 65 °C for 17 h using Agilent Hybridization oven (Agilent Technology, CA, USA). The hybridized microarrays were washed according to the manufacturer’s washing protocol (Agilent Technology, CA, USA). We performed global gene expression analyses using Agilent’s Mouse Gene Expression 4X44K v2 Oligo Microarray (Agilent Technology, CA, USA) (39,429 probes). All raw data sets from the microarray experiments are available at NCBI GEO database under the accession number GSE79109 (Data Citation 2). The experimental design for the microarray analysis process is illustrated in Fig. 1b.

### Microarray data acquisition and analysis

#### Normalization

The hybridized images were scanned using Agilent’s DNA microarray scanner and quantified with Feature Extraction Software (Agilent Technology, CA, USA). Raw intensity data was globally normalized as described previously^23^. Normalized ratios were calculated by dividing the average of normalized intensity in test samples by the average of normalized intensity in control samples. Unpaired *t*-test was performed for statistical analysis. In addition, a q-value was obtained by Significant Analysis of Microarray (SAM) for multiple test correction. Z normalization and tests for significant changes were performed as previously described^23^. Raw data were subjected to Z normalization to ensure compatibility using the formula:

z(raw data)={ln(raw data)−avg[ln(raw data)]}/{s.d.[ln(raw data)]},

where ln is natural logarithm, avg is the average over all genes of an array, and std dev is the s.d. over all genes of an array. The Z ratio (between treatment A and B) is given by z(A)—z(B)/s.d. Individual genes with Z ratio>1.5 (*P* value<0.05), and avg intensity>0 were considered statistically significant. Fold change and Z ratio raw data were deposited at figshare (Data Citation 1).

#### qRT-PCR

For quantitative real-time PCR, total RNA from each liver sample was isolated with RNeasy Mini kit according to the manufacturer’s protocol (Qiagen, Limburg, Netherlands) and was reverse transcribed using Superscript II RT (Invitrogen, CA, USA). Real–time RT-PCR was used to analyze mRNA expression (*n*=3 for each group) using the StepOnePlus (Applied Biosystems, CA, USA). Quantification was performed using the ∆∆CT method^24^. The housekeeping gene, glyceraldehyde-3-phosphate dehydrogenase, was used for internal normalization. Samples related to data sets in qRT-PCR are listed in Table 1. The primer sequences are listed in Table 3. Validation of the microarray results by quantitative real-time PCR are shown in the Table 4. The qRT-PCR raw data was deposited at figshare (Data Citation 1).

## Data Records

### Data record 1

Raw data from blood serum analyses of mice treated with resveratrol (RS), fed with Dongjin rice (DJ), or the resveratrol-enriched rice DJ526 (DJ526), the raw data for mouse behavioral tests of RS, DJ, and DJ526 groups, the raw data for qRT-PCR results of the livers of the mice of RS, DJ and DJ526 groups compared with Ctrl groups, Globally normalized data (Fold change raw data file), Z transformed data (Z-ratio raw data file), and the ARRIVE guidelines Checklist were deposited at figshare (Data Citation 1).

### Data record 2

In this study, we performed global gene expression analyses using Agilent’s Mouse Gene Expression 4X44K v2 Oligo Microarray (Agilent Technology, CA, USA) (39,429 probes). The data have been deposited in NCBI’s GEO (http://www.ncbi.nlm.nih.gov/geo/) and are accessible through GEO series accession number GSE79109 (Data Citation 2).

## Technical Validation

### Validation of the microarray data by quantitative real-time PCR (qRT-PCR)

**Figure 2:**
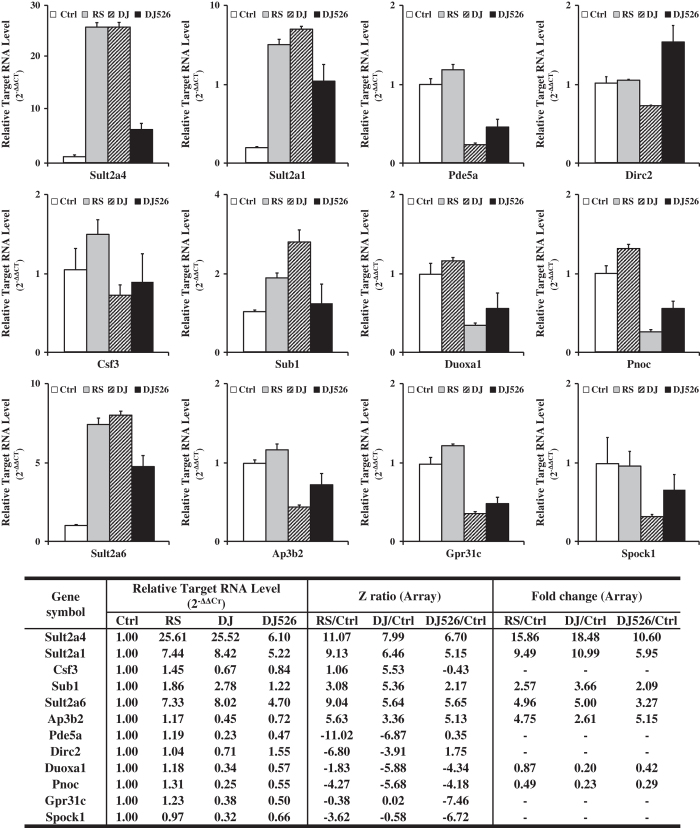
Quantitative real-time PCR data from the mice treated with resveratrol (RS), fed with Dongjin rice (DJ), resveratrol-enriched rice DJ526 (DJ526), and control (Ctrl) diet. Total RNA from the liver samples of Ctrl, RS, DJ, and DJ526 groups was amplified using the primers specific for the indicated targets.

We performed whole-genome microarray and biosynthetic pathway analyses using liver samples of the mice fed with the resveratrol-enriched rice DJ526, Dongjin rice, treated with resveratrol alone, and control. To verify the validity of our microarray data on transcriptional differences, qRT-PCR was performed using RNA from the same liver samples as the microarray analyses, in addition to studying the genes as determined by the microarray analyses. As can be seen in Fig. 2 and Table 4, there is a good correlation between results obtained with the microarray data and those using qRT-PCR. Patterns of gene expression and differences in them were replicated, indicating that this is not a statistical artifact. Broadly speaking, gene expression changes could be grouped into lower expression of *Sult2a4, Sult2a1, Sub1,* and *Sult2a6* in DJ526 than that in DJ and RS. Several genes such as *Pde5a, Csf3, Duoxa1, Pnoc, Ap3b2, Gpr31c,* and *Spock1* are commonly regulated by DJ and DJ526, but not by resveratrol.

## Additional Information

**How to cite this article**: Chung, H.-J. *et al.* Gene expression profiles and physiological data from mice fed resveratrol-enriched rice DJ526. *Sci. Data* 3:160114 doi: 10.1038/sdata.2016.114 (2016).

**Publisher’s note**: Springer Nature remains neutral with regard to jurisdictional claims in published maps and institutional affiliations.

## Supplementary Material



## Figures and Tables

**Table 1:** Samples related to data sets in microarray and qRT-PCR (RNA-extraction quality).

**Sample ID**	**Gender**	**Age**	**Conc. (ng μl^−1^)**	**OD**_**260/280**_	**OD**_**260/230**_	**Result**
Ctrl-1	female	6 month	1,185.9	1.90	2.30	Pass
Ctrl-2	female	6 month	1,501.3	1.89	2.27	Pass
Ctrl-3	female	6 month	1,650.1	1.90	2.27	Pass
DJ-1	female	6 month	1,135.1	1.82	2.34	Pass
DJ-2	female	6 month	1,222.6	1.93	2.23	Pass
DJ-3	female	6 month	1,235.6	1.90	2.31	Pass
RS-1	female	6 month	1,186.6	1.91	2.31	Pass
RS-2	female	6 month	1,072.8	1.80	2.37	Pass
RS-3	female	6 month	1,432.3	1.99	2.17	Pass
DJ526-1	female	6 month	1,459.5	1.87	2.29	Pass
DJ526-2	female	6 month	1,495.9	1.97	2.21	Pass
DJ526-3	female	6 month	1,052.2	1.87	2.37	Pass

**Table 2:** Samples related to data sets in microarray (cRNA-labeling efficiency).

**Sample ID**	**Cy3 (pmol μl^−1^)**	**Constant**	**cRNA Con. (ng μl^−1^)**	**OD**_**260/280**_	**Yield (μg)**	**Specific Activity (total)**
Ctrl-1	4.59	40	278.5	2.17	8.355	16.48
Ctrl-2	4.74	40	285.1	2.20	8.553	16.63
Ctrl-3	4.99	40	301.1	2.16	9.033	16.57
DJ-1	3.95	40	250.6	2.19	7.518	15.76
DJ-2	4.37	40	273.9	2.16	8.217	15.95
DJ-3	4.96	40	289.1	2.14	8.674	17.15
RS-1	4.60	40	284.0	2.18	8.521	16.20
RS-2	4.79	40	294.2	2.21	8.825	16.28
RS-3	4.43	40	280.3	2.17	8.410	15.80
DJ526-1	4.05	40	251.9	2.19	7.558	16.08
DJ526-2	4.52	40	288.7	2.18	8.660	15.66
DJ526-3	4.66	40	289.4	2.16	8.683	16.10
Constant: RNA (40) as the sample type.						
$\mathrm{Yield}:\frac{\left(\text{Concentration}\;\mathrm{of}\;\text{cRNA})\times 30µl\;(\text{Elution}\;\text{volume}\right)}{1000}=µg\;\mathrm{of}\;\mathrm{cRNA}$.						
$\mathrm{Specific activity}:\frac{\mathrm{Concentration}\;\mathrm{of}\;\mathrm{Cy3}}{\mathrm{Concentration}\;\mathrm{of}\;\mathrm{cRNA}}\times 1000=\text{pmol}\;\mathrm{Cy3}\;\mathrm{per}\;µg\;\mathrm{cRNA}$.						

**Table 3:** List of primer used for quantitative real-time PCR.

**Primer name**	**Accession No.**	**Nucleotide Sequence (5′→3′)**	**5′ position**	**Target length**
Sult2a4-F	NM_001101534.1	TCCAGGGTCACTCAGAACTT	423	72
Sult2a4-R	NM_001101534.1	TGCTCAAACCATGATCCATA	494	72
Sult2a1-F	NM_001111296.2	CCAGGGTCACTCGGAACTTA	481	70
Sult2a1-R	NM_001111296.2	GCTCAAACCATGATCCGAAT	550	70
Pde5a-F	NM_153422.2	CGGCCCAAACCCTTAAAATT	1,707	70
Pde5a-R	NM_153422.2	AGCGCTGTTTCCAGATCAGA	1,776	70
Dirc2-F	NM_153550.3	GGCCCCAAAGAAGTAAATGC	4,349	70
Dirc2-R	NM_153550.3	GGTGTGTGGAGGCGATAAGC	4,418	70
Csf3-F	NM_009971.1	AGGAACGAAGTCCCTAAAGAA	1,139	77
Csf3-R	NM_009971.1	CAGGGCCCTAAAAAAGGAAT	1,215	77
Sub1-F	ENSMUST00000110504	CGAGACTTTGGATCCGTGTT	85	74
Sub1-R	ENSMUST00000110504	CTGCCTGAAGAGCTTGAAGA	158	74
Duoxa1-F	NM_145395.2	CATTCCTCTGCTGGCTACTG	645	70
Duoxa1-R	NM_145395.2	AGCATGTGGCCACCATAAAC	715	70
Pnoc-F	NM_010932.2	GCCCCATCTTCTCACTCATC	1,170	70
Pnoc-R	NM_010932.2	CCCAGGTCTGATTTCATGTT	1,239	71
Sult2a6-F	NM_001081325.2	GGAAAAATTTAGGGCCAGAT	645	71
Sult2a6-R	NM_001081325.2	GTTTTCTTTCATGGCTTGGA	715	71
Ap3b2-F	NM_021492.3	CCTCCCATGGTTGTGTCTAC	2,775	74
Ap3b2-R	NM_021492.3	GGCACCAGAGAGGAGTCTGT	2,848	74
Gpr31c-F	NM_001013832.2	TCATCTGGCTTCTGATGGTT	410	72
Gpr31c-R	NM_001013832.2	CCGTGGAATTCTGGGTAGTC	481	72
Spock1-F	NM_009262.3	ATTCGAATTCCCCTTCTTGA	3,147	72
Spock1-R	NM_009262.3	CACCGGGTATATTCATTTCC	3,218	72
GAPDH-F	NM_008084.3	GGCATTGCTCTCAATGACAA	1,132	95
GAPDH-R	NM_008084.3	ATGRAGGCCATGAGGTCCAC	1,226	95
F, forward; R, reverse.				

**Table 4:** Validation of the microarray results by qRT-PCR.

**Gene symbol**	**Relative Target RNA Level (2**^**−ΔΔCт**^)				**Z ratio (Array)**			**Fold change (Array)**		
	**Ctrl**	**RS**	**DJ**	**DJ526**	**RS/Ctrl**	**DJ/Ctrl**	**DJ526/Ctrl**	**RS/Ctrl**	**DJ/Ctrl**	**DJ526/Ctrl**
Sult2a4	1.00	25.61	25.52	6.10	11.07	7.99	6.70	15.86	18.48	10.60
Sult2a1	1.00	7.44	8.42	5.22	9.13	6.46	5.15	9.49	10.99	5.95
Pde5a	1.00	1.19	0.23	0.47	−11.02	−6.87	0.35	0.07	0.39	1.09
Dirc2	1.00	1.04	0.71	1.55	−6.80	−3.91	1.75	0.55	0.19	1.75
Csf3	1.00	1.45	0.67	0.84	1.06	5.53	−0.43	1.53	3.98	1.13
Sub1	1.00	1.86	2.78	1.22	3.08	5.36	2.17	2.57	3.66	2.09
Duoxa1	1.00	1.18	0.34	0.57	−1.83	−5.88	−4.34	0.87	0.20	0.42
Pnoc	1.00	1.31	0.25	0.55	−4.27	−5.68	−4.18	0.49	0.23	0.29
Sult2a6	1.00	7.33	8.02	4.70	9.04	5.64	5.65	4.96	5.00	3.27
Ap3b2	1.00	1.17	0.45	0.72	5.63	3.36	5.13	4.75	2.61	5.15
Gpr31c	1.00	1.23	0.38	0.50	−0.38	0.02	−7.46	1.04	0.96	0.11
Spock1	1.00	0.97	0.32	0.66	0.35	0.20	0.95	1.18	1.00	1.43
The targets were selected based on the Z scores obtained by the microarray analyses, which were provided for the purposes of comparison. Note that Z score numbers reflect statistical confidence and do not relate directly to fold-change.										
